# Human iPSC-Derived Spinal Neurons Carrying the ALS FUS (P525L) Mutation Exhibit Lower Response to Inhibitory Neurotransmitters

**DOI:** 10.1007/s10571-026-01773-z

**Published:** 2026-07-10

**Authors:** Tiziano D’Andrea, Maria Cristina Benedetti, Michela Mochi, Valeria De Turris, Alessandro Rosa, Sergio Fucile

**Affiliations:** 1 IRCCS Neuromed, Pozzilli, IS Italy; 2 Department of Biology and Biotechnologies “Charles Darwin”, Sapienza University of Rome, Rome, Italy; 3 Center for Life Nano- & Neuro-Science, Fondazione Istituto Italiano di Tecnologia (IIT), Rome, Italy; 4 Department of Molecular Medicine, Sapienza University of Rome, Rome, Italy; 5 Department of Physiology and Pharmacology, Sapienza University of Rome, Rome, Italy

**Keywords:** Neurodegeneration, Neurotransmission, Inhibitory/excitatory balance, Glycinergic transmission, Excitotoxicity

## Abstract

Amyotrophic lateral sclerosis (ALS) is a progressive neuromuscular disorder characterized by motoneurons degeneration. Functional studies have linked ALS to hyperexcitability and excitotoxicity, but the cause of the disease is unknown, though familial ALS cases are linked to pathogenic variants in several genes, including *SOD1*, *TARDBP* and *FUS*. Here we focused on the effect of the severe FUS (P525L) mutation on the functional properties of human spinal neurons derived from induced pluripotent stem cells (hiPSCs). This mutation delayed functional maturation, as revealed by the observation that mutated neurons showed alterations of membrane potential, reduced spontaneous synaptic activity, and altered action potentials at early differentiation stages. FUS (P525L) mutation was associated with a significant alteration of inhibitory signalling transmission: mutated neurons showed a significantly lower current response to GABA and glycine compared to control isogenic WT neurons of the same age. Also, glutamatergic currents exhibited a different temporal evolution in control and mutated neurons, but at a lower extent in comparison to inhibitory neurotransmitters. The decrease in the glycine-evoked currents was confirmed by the reduction of the expression of the α1 subunit of glycine receptor, measured by immunofluorescence assay. Similar functional alterations were measured in spinal neurons differentiated form a second hiPSC line, confirming the causative role of the FUS (P525L) mutation. Our data indicate that the FUS (P525L) mutation reduces the maturation rates and the function of hiPSC-derived spinal neurons, with a strong decrease of inhibitory transmission, which may affect the excitatory/inhibitory balance, possibly predisposing to excitotoxicity and neurodegeneration.

**Graphical Abstract:**

**Figure:**
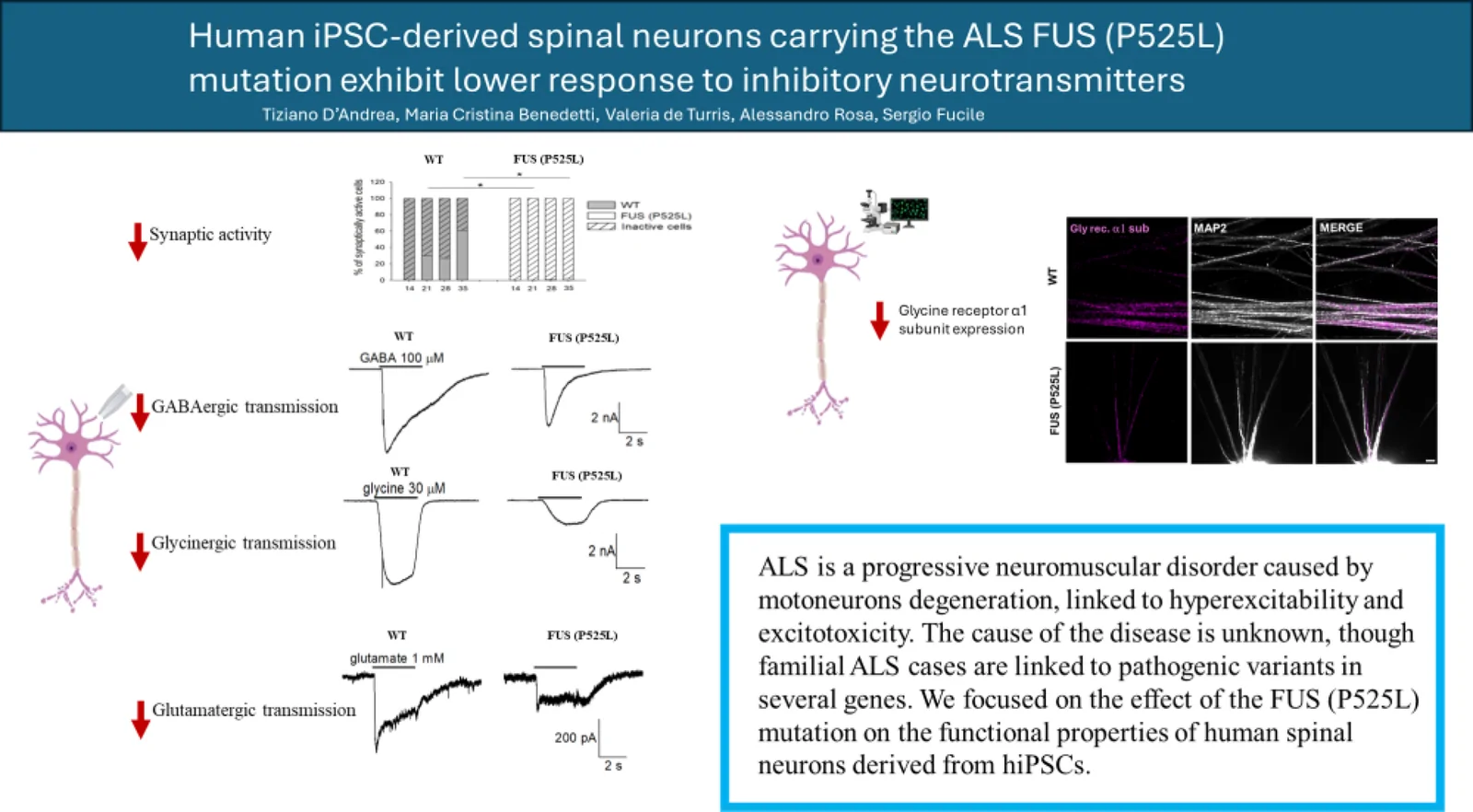

**Supplementary Information:**

The online version contains supplementary material available at https://doi.org/10.1007/s10571-026-01773-z.

## Introduction

Spinal motoneurons, located in the ventral horn of the spinal cord, control effector muscles in the periphery, forming motor units, facilitating co-activation of synergistic muscles, and enabling reciprocal inhibition of antagonistic muscles (Stifani 2014; Benarroch 2021; Taitano et al. 2024). Research on the function of human spinal motoneurons is challenging due to the technical and ethical difficulty to obtain these cells. Human induced pluripotent stem cells (hiPSCs), obtained from accessible patient tissue samples (such as fibroblasts, blood cells, epithelial cells, and keratinocytes), are now widely used for in vitro disease modeling (De Santis et al. 2018; Amorós et al. 2022) and can be converted in spinal motoneurons to study several diseases, including amyotrophic lateral sclerosis (ALS). ALS is a progressive neuromuscular disorder characterized by brain and spinal cord motoneurons degeneration, which in a few years spreads involving most muscles, including the diaphragm (Brown and Al-Chalabi 2017). The cause of the disease is largely unknown, but about 20% of familial ALS cases are caused by various mutations in the superoxide dismutase 1 (*SOD1*) gene; variants in TAR DNA-bnding protein 43 (*TARDBP*), account for an additional 5% of familial cases; mutations in the fused in sarcoma (*FUS*) gene, which also encodes a DNA/RNA binding protein, have ben identified in 5% of patients with familial ALS (Ticozzi et al. 2009). The FUS protein is involved in transcription, splicing, microRNA processing, translatin and shuttling of RNA from the nucleus to the cytoplasm and is also a transcriptional regulatory sensor of DNA damage signals, important in maintaining genome integrity; in the CNS, it regulates the morphology of dendritic spines and stabilizes the structure of the synapse (Ticozzi et al. 2009). Hyperexcitability, an increased propensity of neurons to generate an action potential in response to stimuli, has been extensively described in both ALS patients and animal models (Cheng et al. 2024). Hyperexcitability leads to an excessive release of glutamate, causing severe damage and death of neurons in response to an excessive Ca^2+^ influx through the glutamate receptors (Odierna et al. 2024). Thus, excitotoxicity is a therapeutic target for ALS treatments: riluzole, which reduces glutamatergic currents, is one of only three approved small-molecular drugs for ALS (Bensimon et al. 1994). ALS is also connected to glutamate excitotoxicity due to the impairment of its uptake by astrocytes, leading to an increased influx of Ca^2+^ ions in the motoneurons through NMDA receptors (Bonafede and Mariotti 2017). The resulting high [Ca^2+^]_i_, is not properly coped due to mitochondrial dysfunctions, and causes neurodegeneration activating Ca^2+^-dependent enzymatic pathways contributing to oxidative stress. This process is exacerbated by intracellular aggregates formed by misfolded proteins (such as SOD1, TDP-43 and FUS), and by activated astrocytes and microglia releasing inflammatory mediators and toxic factors (Bonafede and Mariotti 2017). A consolidated finding in the study of ALS-related functional mechanisms is the observed reduction of inhibitory signalling (Tessitore et al. 2026). ALS subjects show the reduction of inhibitory interneurons in the ventral and intermediate areas of the spinal cord, confirmed in presymptomatic SOD1 (G93A) mice, a mutation that causes a preferential loss of inhibitory inputs on motoneurons (Oyanagi et al. 1989; Mora and Allodi 2023). Murine models of ALS show a reduction of glycinergic current densities, along with a significant decrease of the glycinergic synaptic inputs onto motoneurons in both presymptomatic and symptomatic mice (Chang and Martin 2009, 2011; Salamatina et al. 2020). Degeneration occurs only at later stages of ALS progression intraspinal excitatory interneurons: excitatory synaptic inputs on spinal motoneurons are reduced in several mouse ALS models (Qiu et al. 2014; Romer et al. 2017; Salamatina et al. 2020; Broadhead et al. 2022). More recently, an altered motoneuronal function has been reported by several groups using hiPSC-derived spinal motoneurons with ALS-related mutations, finding altered excitability and synaptic transmission (Wainger et al. 2014; Devlin et al. 2015; Yang et al. 2023). In this context, we aim to study the function of human spinal motoneurons derived from hiPSCs carrying the FUS (P525L) mutation, with a particular focus on the ability of these cells to respond to different neurotransmitters, to better understand the mechanisms underlying ALS onset in humans.

## Methods

### hiPSCs Culture and Differentiation

In this paper we have used isogenic FUS P525L mutant and wild-type hiPSCs lines described in (Lenzi et al. 2015; Marrone et al. 2018). Cells have been cultured in Nutristem-XF (Biological Industries) containing 0.1× Penicillin/Streptomycin (Merck Life Sciences) (Nutristem + P/S), changed daily, on dishes coated with hESC-qualified Matrigel (Corning). Cells were dissociated in 1 mg/mL Dispase II (Thermo Fisher Scientific) and passaged every 4–5 days. Mycoplasma tests were run routinely to exclude contamination. For motoneuron differentiation, cells were first stably modified by introducing the Ngn2-Isl1-Lhx3 module (NIL) encoded in a piggyBac vector (De Santis et al. 2018) and then induced to differentiate as described (Garone et al. 2019). Briefly, single cells obtained by dissociation with Accutase (Thermo Fisher Scientific), were seeded in Nutristem + P/S supplemented with 10 µM Y-27,632 (Enzo Life Sciences) at a concentration of 500.000 cells in 35 mm dishes. The day after, medium was switched to DMEM/F12 (Merck Life Sciences), 1× Glutamax, 1× NEAA, 0.5× P/S (all from Thermo Fisher Scientific). At this time point 1 µg/mL doxycycline was added to induce NIL expression. From day 3 to day 5 cells were cultured in Neurobasal/B27 medium (Neurobasal; 1× B27, 1× Glutamax, 1× NEAA, 0.5× P/S; all from Thermo Fisher Scientific), containing 1 µg/mL doxycycline, 5 µM DAPT, 4 µM SU5402 (both from Merck Life Sciences). At day 5, neuronal progenitor cells dissociated with Accutase were plated in each 35 mm Matrigel-coated dish in Neurobasal/B27 medium in presence of 10 µM Y-27,632 at a concentration of 4 × 10⁶ in 35 mm dishes. From the following day cells were maintained in Neurobasal/B27 medium, 20 ng/mL L-ascorbic acid (Merck Life Sciences), 20 ng/mL BDNF, and 10 ng/mL GDNF (both from PeproTech) and the analysis were performed after 21 days of cell culture. Each differentiation batch is considered here as a biological replicate. Three different differentiation batches were used for each functional experiment.

### Electrophysiology

Whole-cell patch-clamp recordings were performed blindly on hiPSC-derived spinal neurons after 14, 21, 28, and 35 days of differentiation (Day in Vitro, DiV). Cells were plated in 35 mm Petri-dishes and recorded at room temperature (25 °C). Membrane currents and voltage were recorded using glass electrodes (3–4 MΩ) filled with 140 mM KCl, 10 mM Hepes, 5 mM BAPTA, and 2 mM Mg-ATP (pH 7.3, with KOH). During recording, cells were continuously superfused using gravity-driven perfusion tubes connected to a fast exchanger system (RSC-160; Bio-logic, Claix, France). The external solution (NES) was composed of: 140 NaCl, 2.8 KCl, 2 CaCl_2_, 2 MgCl, 10 glucose, and 10 HEPES; pH value adjusted with NaOH 1 M at pH 7.3. The identification of neurons followed morphological criteria: highly birefringent cells with small diameter processes. Several agonists were delivered to cells while recording whole-cell currents at −70 mV membrane potential (voltage clamp): GABA 100 µM, glutamate (glut) 1 mM, glycine (gly) 30 µM, acetylcholine (ACh) 100 µM. Action potentials (APs) were recorded in current-clamp configuration applying depolarizing current steps (4–50 pA, 1 s). For the relative experiments, 100 nM TTX was used. Membrane potentials and currents were acquired at 50 kHz and filtered at 3 kHz with a HEKA EPC 800 amplifier (HEKA Elektronik, Reutlingen, Germany), a Digidata 1322 A analog-to-digital converter, and pClamp 10 software (Molecular Devices, Union City, CA; RRID: SCR_021041 and RRID: SCR_011323), and analysed off-line.

### Immunofluorescence

hiPSC-derived neuronal cells cultured in mSlide 8 well (ibidi) for 21 days were fixed in 4% paraformaldehyde (PFA) and 30 mM sucrose for 15 min at room temperature, then washed with phosphate-buffered saline containing calcium and magnesium (PBS +/+, Sigma-Aldrich), and incubated 15 min in PBS (Merck Life Sciences) containing 0.5% BSA, 10% horse serum (HS), 0.2% Triton X-100 (Merck Life Sciences) for permeabilization. Cells were then washed twice in PBS and blocked for 30 min in PBS containing 0.5% BSA, 10% HS, 0.2% Triton X-100 (blocking solution). Incubation time for primary antibodies (diluted in blocking solution) was overnight at 4 °C except for anti-MAP2, which was used for 1 h at room temperature. After two washes in PBS, cells were incubated for 1 h at room temperature with secondary antibodies. Following PBS washes, cells were mounted in ibidi Mounting Medium (50001, ibidi).

Primary antibodies used in this study were: chicken anti-MAP2 (ab5392, Abcam; RRID: AB_2138153) at a dilution of 1:2000, rabbit anti-glycine receptor a1 at a dilution of 1:50 (PA5-102668, Invitrogen; RRID: AB_2852065), rabbit anti-GluA1 at a dilution of 1:100 (SAB4501294, Merck Life Sciences; RRID: AB_10745377), anti-BASSOON at a dilution of 1:500 (ab82958, Abcam; RRID: AB_1860018), anti-VGAT at a dilution of 1:1500 (131003, SYSY Antibodies; RRID: AB_887869), anti-CHAT at a dilution of 1:1200 (ma5-31383, Thermo Fisher Scientific; RRID: AB_2787020), anti-GADD67 at a dilution of 1:150 (sc-28376, Santa Cruz Biotechnology; RRID: AB_627650). Alexa Fluor–conjugated secondary antibodies against mouse (A647; A32728, Thermo Fisher Scientific; RRID: AB_2866490), chicken (A488; IS20020, Immunological Sciences; RRID: AB_10559185), and rabbit (A594; IS20152-1, Immunological Sciences; RRID: AB_10853603) were used at a dilution of 1:250. Nuclei were counterstained with DAPI (Merck Life Sciences). 15 fields were acquired for each replicate. Image analysis was performed with ImageJ (Fiji; RRID: SCR_002285) using the ‘Analyze Particles’ function for the quantification of Glycine receptor a1 puncta, and manual counting for GluA1 puncta. Puncta counts were normalized to the MAP2-positive area, and the mean value was then calculated for each biological replicate.

Image stacks were acquired using a 20× objective with an inverted Olympus iX73 microscope equipped with an X-Light V3 spinning disc head (Crest Optics), an LDI laser illuminator (89 North), and a Prime BSI sCMOS camera (Photometrics), using 405 nm, 470 nm, 555 nm, and 640 nm excitation wavelengths. Acquisition was performed with MetaMorph software (Molecular Devices; RRID: SCR_002368). Image stacks used in figure W and Z were acquired using a 20× objective with an inverted Nikon Ti2 microscope equipped with an X-Light V3 spinning disc head (Crest Optics), an LDI laser illuminator (89 North), and a Prime BSI sCMOS camera (Photometrics), using 405 nm, 470 nm, 555 nm, and 640 nm excitation wavelengths. Acquisition was performed with a NIS-Element AR software (Nikon; RRID: SCR_027181). Spot density was quantified using ImageJ by counting the number of receptor marker-positive puncta colocalizing with the TUJ-positive area. This value was normalized to the total TUJ-positive area. For each biological replicate, 7–8 fields of view were analyzed, and the measurements were averaged to obtain a single value per replicate.

### Statistics

All data were expressed as means ± S.D. and analysed using one-way ANOVA or paired t-test, as appropriate. Tests for normality and variance heterogeneity were conducted on each data set. When necessary, the non-parametric Dunn’s one-way ANOVA on ranks was used. In case of significance, all pairwise multiple comparison procedure was used (Holm-Sidak, or Dunn’s method for non-parametric tests). The minimum power of statistical tests was set at 0.8. The significance for all tests was set at *p* < 0.05.

## Results

### Molecular Characterization of Neuronal Cultures

**Fig. 1:**
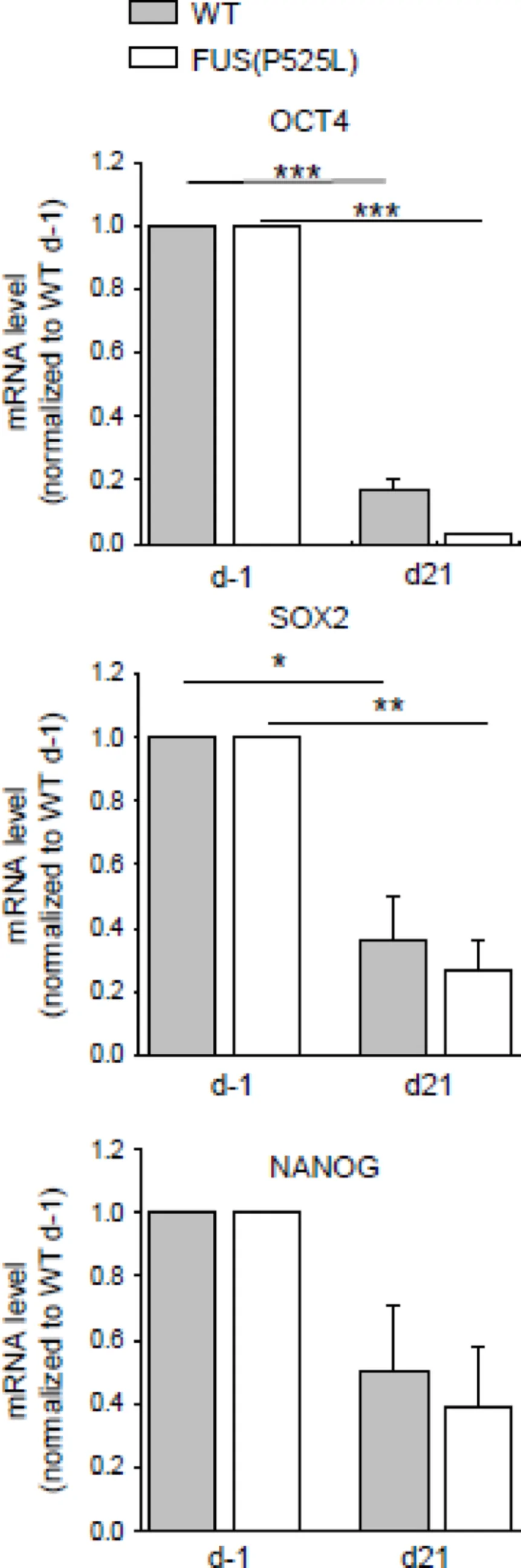
Quantitative analysis of pluripotence markers. mRNA levels of *OCT4* (top), *SOX2* (center) *and NANOG* (bottom) in WT and FUS (P525L) hiPSCs (indicated as d-1) and neural progenitors at day 5 of differentiation (*: *p* = 0.02, **: *p* = 0.03; ***: *p* < 0.001). ATP5O expression was used for normalization, and the graphs show the average, standard deviation, and *p*-values from 3 replicates, each consisting of a batch of differentiated hiPSCs. Two-way ANOVA and post hoc Tukey test p-values for multiple comparisons have been used

**Fig. 2:**
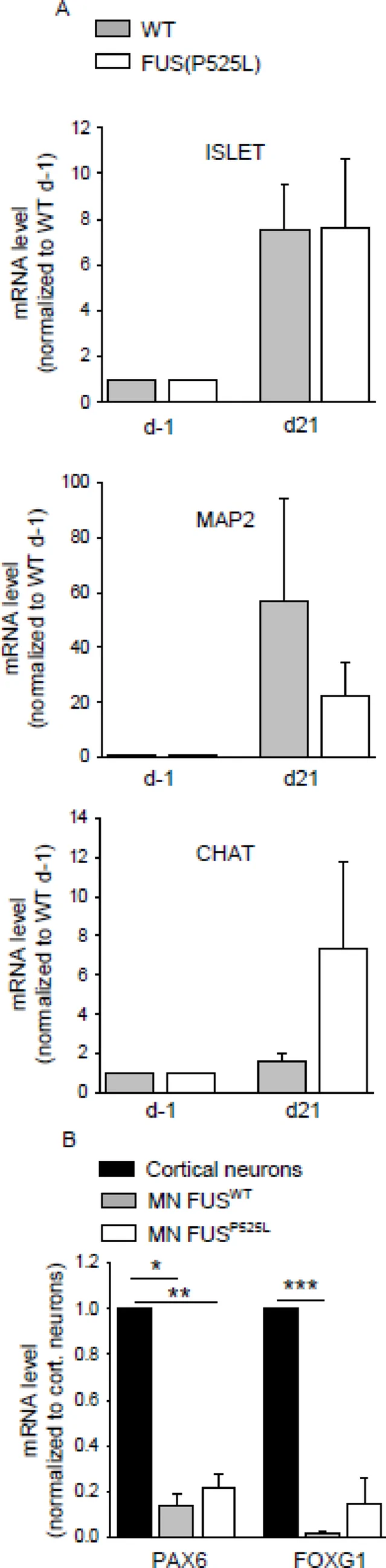
Quantitative analysis of motoneuronal markers. **A**, mRNA levels of *MAP2* (left), *CHAT* (center) and *ISLET* (right) in WT and FUS (P525L) hiPSCs (indicated as d-1) and spinal neurons at day 21 of differentiation. **B**, quantitative analysis of *PAX6* and *FOXG1* mRNA levels in cortical neurons and WT and FUS (P525L) spinal neurons (*: *p* = 0.0121, **: *p* = 0.0195; ***: *p* < 0.001). ATP5O expression was used for normalization, and the graphs show the average, standard deviation, and *p*-values from 3 replicates, each consisting of a batch of differentiated hiPSCs. Two-way ANOVA and post hoc Tukey test p-values for multiple comparisons are indicated in the graphs

**Fig. 3:**
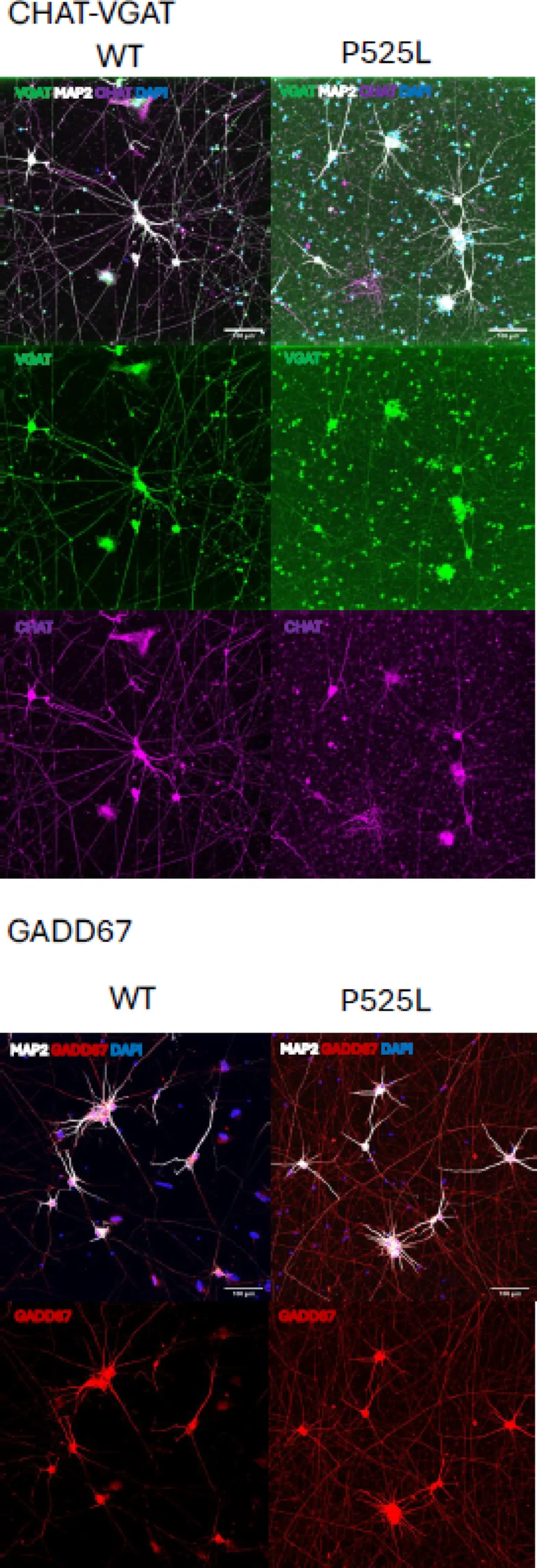
Expression of cholinergic and GABAergic markers. Representative images of immunofluorescence staining of spinal neurons culture at day 21 using the indicated primary antibodies and DAPI. Immunostaining analysis of spinal neurons culture at day 21 of differentiation. MAP2 (gray) is a neuronal marker. DAPI (blue) was used for nuclear staining. Immunostaining analysis of spinal neurons culture at day 21 of differentiation. MAP2 (gray) is used as a neuronal marker. DAPI (blue) was used for nuclear staining

Neuronal cultures with a spinal cord identity were obtained by differentiation of isogenic WT and FUS (P525L) hiPSCs (WT I lines; (Lenzi et al. 2015) as previously described (De Santis et al. 2018; Garone et al. 2019). Differentiated cells were first characterized in terms of downregulation of pluripotency markers and activation of neuronal markers by qRT-PCR. As shown in Fig. 1, in both expression levels of the pluripotency-associated genes *OCT4*, *SOX2* and *NANOG* were decreased at 21 days of differentiation. At the same time point we observed upregulation of the pan-neuronal marker *MAP2* and the motoneuronal markers *ISL1* and *CHAT* (Fig. 2A). To further characterize the identity of the differentiated neurons, we assessed the expression levels of the forebrain markers *PAX6* and *FOXG1* in comparison with those detected in hiPSC-derived cortical neurons (obtained as described in Benedetti et al. 2024). As shown in Fig. 2B, low levels of these anterior markers confirmed the posterior identity of the neuronal populations obtained. Interestingly, immunostaining analysis confirmed the expression of the motoneuron marker CHAT along with GABAergic markers (VGAT- and GADD67-positive cells) at comparable levels in both genotypes (Fig. 3).

Taken together, these results suggest that the neuronal population obtained from WT and P525L mutant FUS hiPSCs have a posterior identity and contain both motoneurons and GABAergic inhibitory neurons.

### FUS (P525L) Mutation Reduces Neuronal Hyperpolarization and Spontaneous Synaptic Activity in hiPSC-Derived Spinal Neurons

**Fig. 4:**
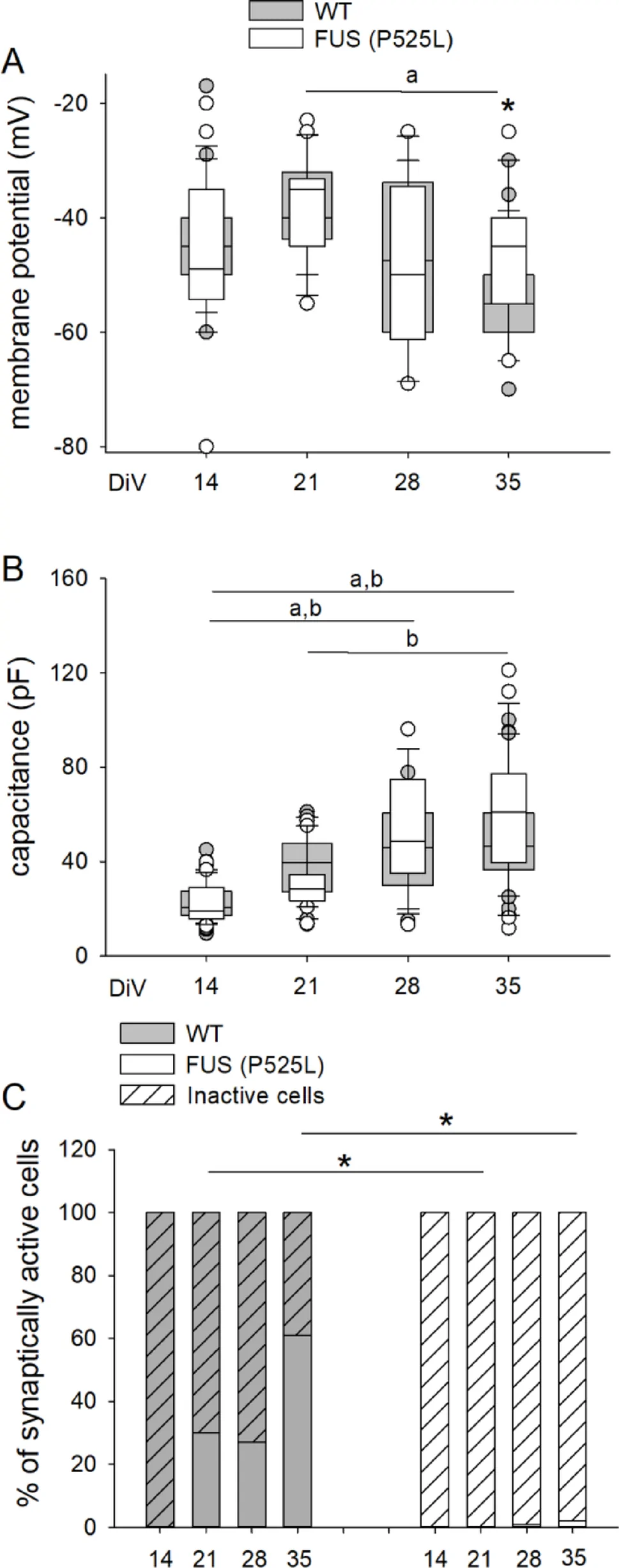
hiPSC-derived FUS (P525L) spinal neuron exhibits reduced synaptic activity compared to WT.**A**, box and whiskers plot showing the changes of the membrane resting potential at the indicated DiV of WT (grey) and FUS (P525L) neurons (white) (a: *p* < 0.001, F_1,37_ = 19.95; *: *p* = 0.012, F_1,43_ = 6.81). **B**, box and whiskers plot showing the cell membrane capacitance of WT (grey) and FUS (P525L) neurons (white) at the indicated DiV (WT DiV 28 vs. DiV 14, a: *r* = 19.560, *p* < 0.001; WT DiV 35 vs. DiV 14, a: *r* = 27.343, *p* < 0.001; FUS (P525L): DiV 28 vs. DiV 14, b: *r* = 16.523, *p* < 0.001; FUS (P525L) DiV 35 vs. DiV 14, b: *r* = 18.726, *p* < 0.001; FUS (P525L) DiV 35 vs. DiV 21, b: *r* = 13.727 *p* = 0.014). **C** percentage of WT (grey) and FUS (P525L) neurons (white) showing spontaneous synaptic activities (DiV 21 *: *p* = 0.025; DiV 35 *: *p* = 0.032). All plots show data obtained at 4 early maturation stages: DiV 14 (n _WT_ = 35, n _FUS(P525L)_ = 27), DiV 21 (n _WT_ = 21, n _FUS(P525L)_ = 22), DiV 28 (n _WT_ = 18, n _FUS(P525L)_ = 16), DiV 35 (n _WT_ = 31, n _FUS(P525L)_ = 22). Recorded cells were obtained from 3 independent differentiation batches, for each time-point. Data were obtained from hiPSC-derived spinal neurons of the WT I cell line. Significance was determined using a one-way ANOVA and attested on a a p value *p* < 0.05

The resting membrane potential, the cell capacitance, and the synaptic function of both the WT and the FUS (P525L) neurons (WT I lines; (Lenzi et al. 2015) were measured at 4 different maturation stages (DiV 14, 21, 28, 35). The WT neurons reached the physiological hyperpolarization typical of neuronal maturation at DiV 35, while FUS (P525L) neurons remained significantly more depolarized (Fig. 4A). By contrast, there was no significative difference in cell capacitance (Fig. 4B), which in both groups showed an increase compatible with cell growth and maturation. The percentage of synaptically active WT cells (Fig. 4C) was higher in all the time-points compared to their FUS (P525L) counterparts. These data indicate a delay in the functional maturation of FUS (P525L) spinal neurons.

### FUS (P525L) Spinal Neurons Show an Early Impairment in Action Potentials Which Reverts During Maturation

**Fig. 5:**
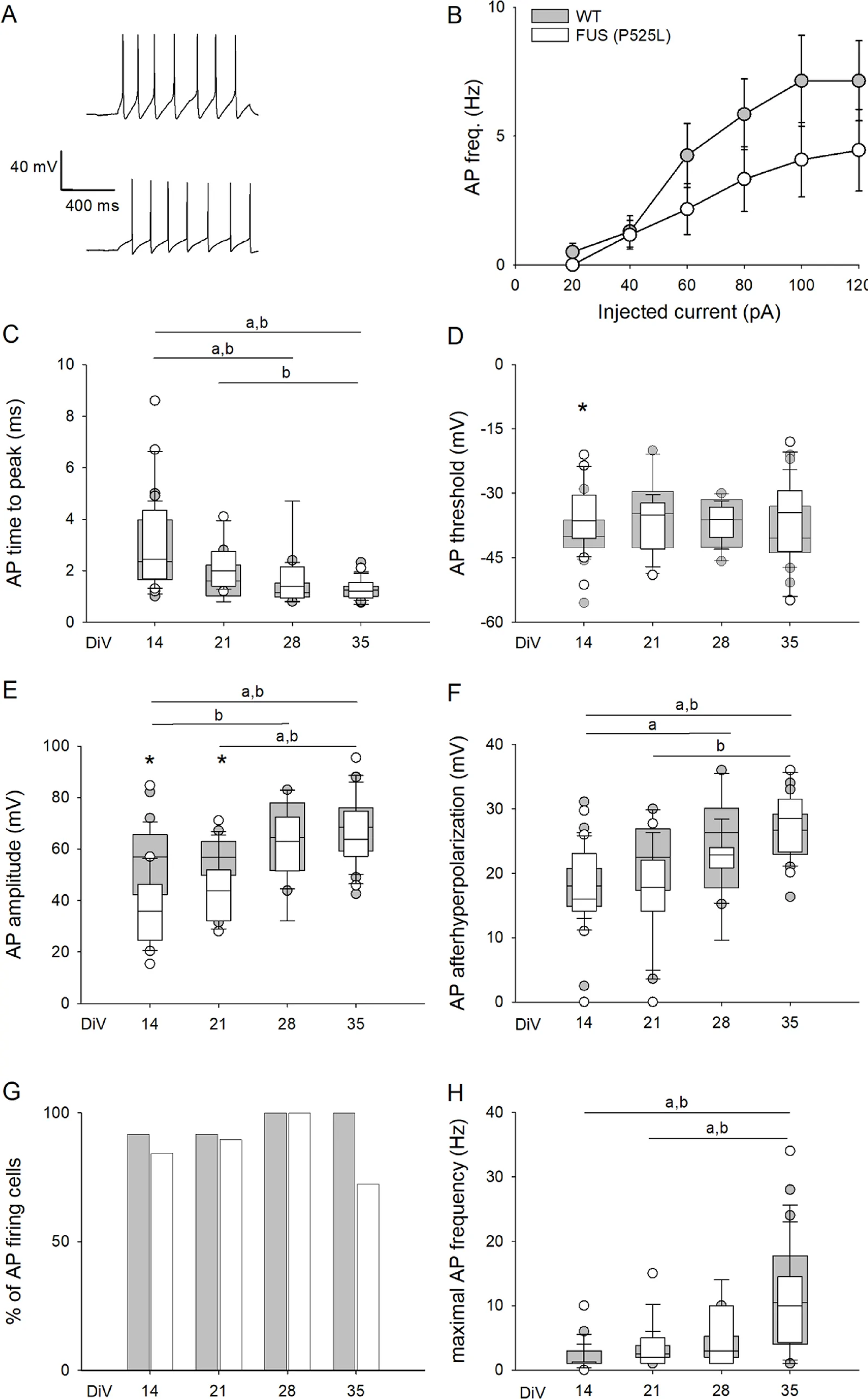
hiPSC-derived WT and FUS (P525L) spinal neurons exhibit similar action potential properties during early maturation stages.**A**, representative evoked APs recorded from WT (top) and FUS (P525L) neurons (bottom), at DiV 28. **B**, Input–output relationship for WT (grey), FUS (P525L) neurons (white), at DIV 35. **C**, time-course of the time to peak (from threshold to peak) of APs evoked from WT (grey) and FUS (P525L) neurons (white); WT: DiV 35 vs. DiV 14: a: *r* = 17.667, *p* < 0.001, DiV 28 vs. DiV 14: a: *r* = 12.396, *p* < 0.001; FUS (P525L): DiV 35 vs. DiV 14: b: *r* = 13.898, *p* < 0.001, DiV 28 vs. DiV 14: b: *r* = 8.700, *p* = 0.011, DiV 35 vs. DiV 21: b: *r* = 10.724, *p* < 0.001). **D**, time-course of the AP threshold of WT and FUS (P525L) neurons (*: F_1,42_ = 4.555, *p* = 0.039). **E**, time-course of the amplitude (from threshold to peak) of APs of WT and FUS (P525L) neurons; WT vs. FUS (P525L) DiV 14: *: *r* = 17.844, *p* < 0.001; WT vs. FUS (P525L) DiV 21: *: F_1,25_ = 5.066, *p* = 0.033; WT DiV 35 vs. DiV 14, a: F_1,46_ = 11.941, *p* = 0.005; WT DiV 35 vs. DiV 21, a: F_1,35_ = 8.153, *p* = 0.038; FUS (P525L) DiV 35 vs. DiV 14, b: *r* = 13.962, *p* < 0.001; FUS (P525L) DiV 28 vs. DiV 14, b: *r* = 23.993, *p* < 0.001; FUS (P525L) DiV 35 vs. DiV 21, b: *r* = 22.533, *p* < 0.001). **F**, time-course of the AP afterhyperpolarization of WT and FUS (P525L) neurons (WT DiV 35 vs. DiV 14, a: F_1,43_ = 31.495, *p* < 0.001; WT DiV 28 vs. DiV 14, a: F_1,29_ = 8.330, *p* = 0.016; FUS (P525L) DiV 35 vs. DiV 14, b: F_1,29_ = 24.955, *p* < 0.001; FUS (P525L) DiV 35 vs. DiV 21, b: F_1,28_ = 21.579 *p* < 0.001). **G**, percentage of WT and FUS (P525L) neurons able to produce APs. **H**, max number of evoked APs per neuron of WT and FUS (P525L) neurons (WT DiV 35 vs. DiV 14, a: *r* = 17.833, *p* < 0.001, WT DiV 35 vs. DiV 21, a: *r* = 10.483, *p* = 0.071; FUS (P525L) DiV 35 vs. DiV 14, b: *r* = 15.006, *p* < 0.001, FUS (P525L) DiV 35 vs. DiV 21, b: *r* = 10.191, *p* = 0.036). All plots show data obtained at 4 early maturation stages: DiV 14, 21, 28, 35, same cells as Fig. 1. Data were obtained from hiPSC-derived spinal neurons of the WT I cell line. Significance was determined using a one-way ANOVA and attested on a p value *p* < 0.05

Action potentials (APs) were evoked from the WT and FUS (P525L) hiPSC-derived neurons, and several parameters were analysed at different maturation stages (Fig. 5A). The relationship between injected current and the APs frequency, measured at DiV 35, did not show any differences between WT and FUS (P525L) neurons (Fig. 5B). In both conditions the APs time to peak was significantly reduced during maturation (Fig. 5C). The APs threshold was unaltered during maturation, showing a significant difference between WT and mutated neurons only in the earliest maturation stage, at DiV 14 (Fig. 5D). APs amplitude showed an impairment in FUS (P525L) neurons during the early maturing stages (Fig. 5E) compared to WT neurons. Anyway, both groups underwent a clear maturation process of the APs machinery, enhancing their APs amplitude during time. APs afterhyperpolarization also showed an increase during neuronal maturation (Fig. 5F), both in WT and FUS (P525L) neurons. Finally, we measured the fraction of neurons able to produce APs (Fig. 5G) and the maximal Aps frequency of APs (Fig. 5H). This last parameter showed an increase of APs firing in the late maturing stages in both WT and FUS (P525L) neurons.

### Impairment of Neurotransmitters Response in hiPSCs-Derived FUS (P525L) Spinal Neurons

**Fig. 6:**
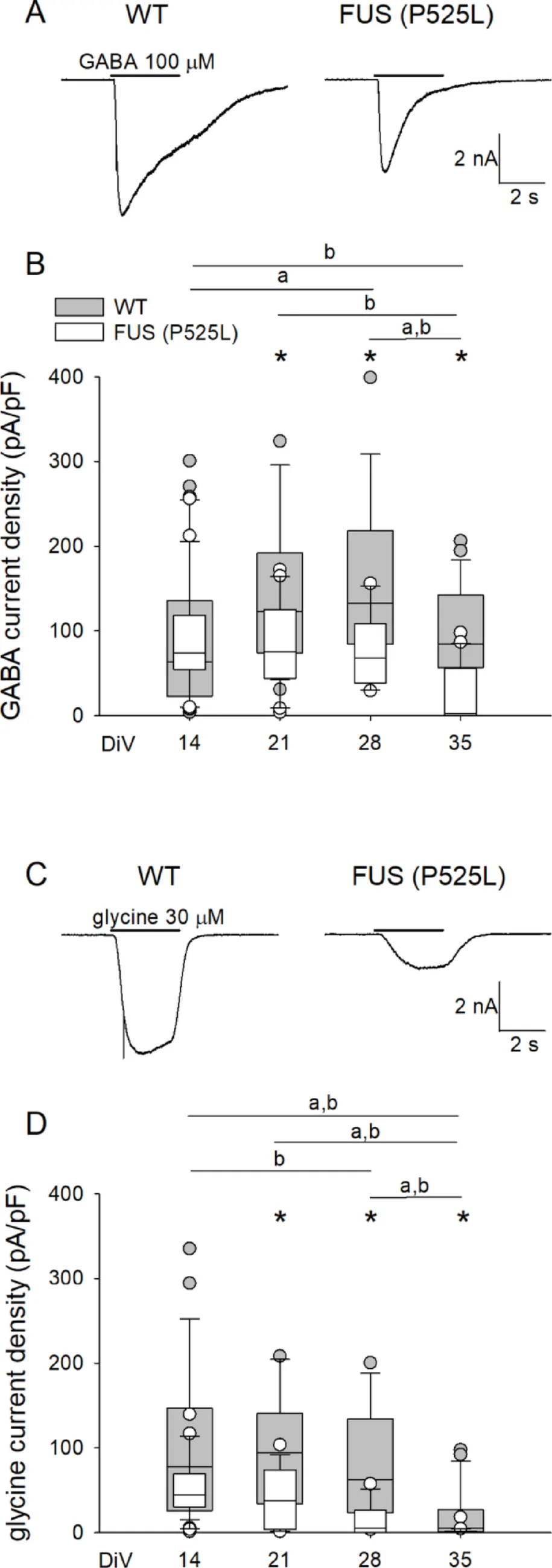
FUS (P525L) mutation reduces the response of hiPSC-derived spinal neurons to inhibitory neurotransmitters.**A**, representative whole-cell currents evoked by GABA (100 mM) recorded from WT (left) and FUS (P525L) neurons (right), at DiV 21. B, time-course of GABA current densities measured from WT (grey) and FUS (P525L) mutated (white; WT vs. FUS (P525L) DiV 21 *: F_1,36_ = 6.549, *p* = 0.015, WT vs. FUS (P525L) DiV 28 *: *r* = 9.107, 549, *p* = 0.005, DiV 35 *: *r* = 19.262, *p* < 0.001; WT DiV 14 vs. DiV 28, a: *r* = 11.560, *p* = 0.016; WT DiV 35 vs. DiV 28, a: *r* = 8.825, *p* = 0.031; FUS (P525L) DiV 35 vs. DiV 14, b: *r* = 15.950, *p* < 0.001; FUS (P525L) DiV 35 vs. DiV 21, b: *r* = 13.300, *p* = 0.002; FUS (P525L) DiV 35 vs. DiV 28, b: *r* = 11.657, *p* = 0.011). C, representative whole-cell currents evoked by glycine (30 mM) recorded from WT (left) and FUS (P525L) neurons (right), at DiV 21. D, time-course of glycine current densities measured from WT and FUS (P525L) mutated neurons (WT vs. FUS (P525L) DiV 21 *: *r* = 7.933, *p* = 0.021; WT vs. FUS (P525L) DiV 28 *: *r* = 9.791, *p* = 0.001; WT vs. FUS (P525L) DiV 35 *: *r* = 14.409, *p* < 0.001; WT DiV 35 vs. DiV 14, a: *r* = 20.147, *p* < 0.001; WT DiV 35 vs. DiV 21, a: *r* = 17.651, *p* < 0.001; WT DiV 35 vs. DiV 28, a: *r* = 17.651, *p* = 0.007; FUS (P525L) DiV 35 vs. DiV 14, b: *r* = 21.429, *p* < 0.001; FUS (P525L) DiV 28 vs. DiV 14, b: *r* = 13.045, *p* < 0.012; FUS (P525L) DiV 35 vs. DiV 21, b: *r* = 17.895, *p* < 0.001). All plots show data obtained at 4 early maturation stages: DiV 14, 21, 28, 35, same cells as Fig. 1. Data were obtained from hiPSC-derived spinal neurons of the WT I cell line. Significance was determined using a one-way ANOVA and attested on a p value *p* < 0.05

**Fig. 7:**
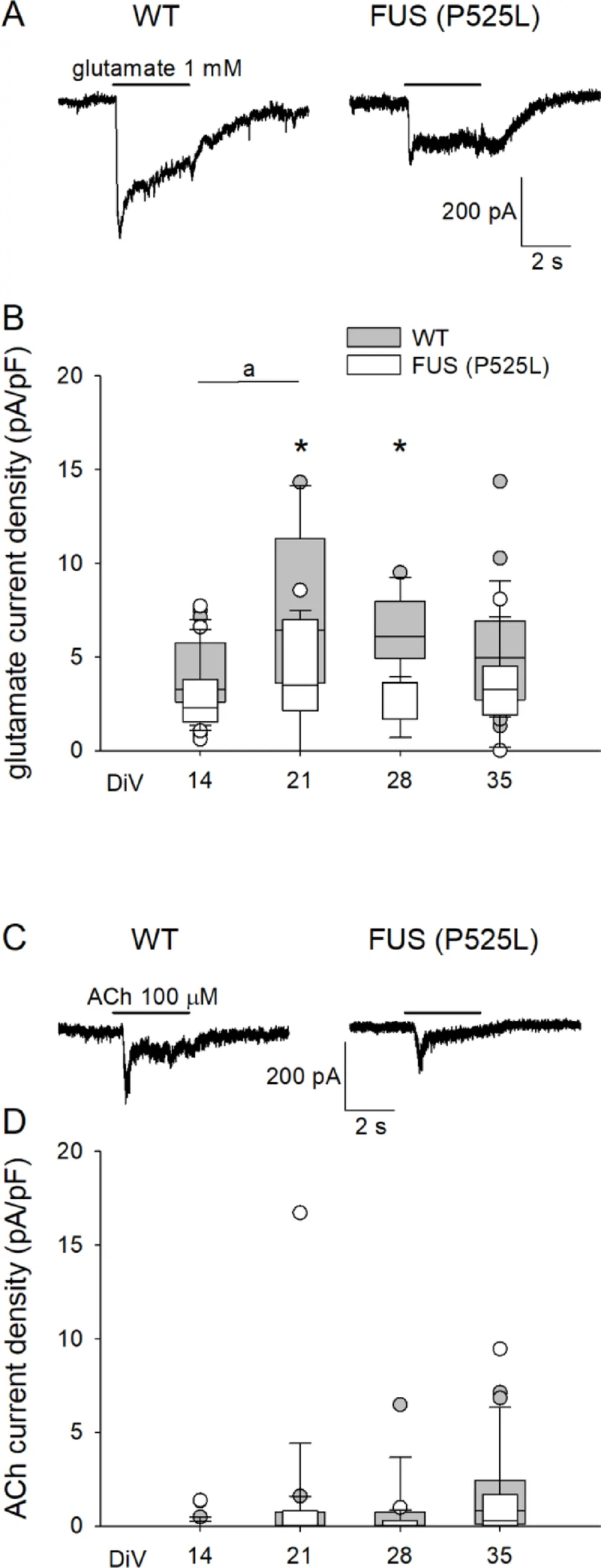
hiPSC-derived FUS (P525L) spinal neurons exhibit a different temporal evolution of glutamatergic currents, compared to WT. **A**, representative whole-cell currents evoked by glutamate (1 mM) recorded from WT (left) and FUS (P525L) neurons (right), at DiV 21. **B**, time-course of glutamate current densities measured from WT (grey) and FUS (P525L) neurons (white; WT vs. FUS DiV 21, *: F_1,32_ = 9.049, *p* = 0.005; WT vs. FUS (P525L) DiV 28, *: F_1,16_ = 14.859 *p* = 0.001; WT DiV 14 vs. DiV 21, a: *r* = 10.315 *p* = 0.012). **C**, representative whole-cell currents evoked by acetylcholine (100 mM) recorded from WT (left) and FUS (P525L) neurons (right), at DiV 21. **D**, time-course of acetylcholine current densities measured from WT and FUS (P525L) mutated neurons. All plots show data obtained at 4 early maturing stages: DiV 14, 21, 28, 35, same cells as Fig. 1. Data were obtained from iPSC-derived spinal neurons of the WT I cell line. Significance was determined using a one-way ANOVA and attested on a p value *p* < 0.05

The current responses to 4 neurotransmitters were recorded from WT and FUS (P525L) hiPSC-derived spinal neurons at 4 maturation stages (DiV 14, 21, 28, 35). GABA responses in WT neurons reached a peak at DiV 28 and then decreased (Fig. 6A, B). In FUS (P525L) neurons GABA responses decreased during maturation, leading to significant differences with WT neurons, indicating an impairment of GABAergic transmission associated with FUS (P525L) mutation. Glycine responses exhibited a decrease during maturation in both WT and mutated neurons, with FUS (P525L) neurons showing a significantly lower glycine response compared to WT neurons of the same age (Fig. 6C). Glutamate responses were stable during maturation in FUS-mutant cells, while in WT cells increased in the earliest stages of maturation (Fig. 7A, B). Thus, glutamatergic responses were lower in FUS (P525L) cells, compared to WT, at DiV 21 and DiV 28, but not at DiV 35. Finally, we administered acetylcholine (Fig. 7C, D), which did not elicit responses in most neurons and showed no differences between WT and mutated neurons. The same reduction in the amplitudes of whole-cell current evoked by neurotransmitters were observed in mutated neurons in the presence of TTX 100 nM (supplementary Fig S1A-C), showing that this functional alteration is not due to changes in neuronal excitability.

To confirm the effect of the FUS (P525L) mutation on neuronal activity, we performed the same procedures on isogenic WT and FUS (P525L) spinal neurons derived from a different hiPSC line (T 12.9 line; Marrone et al. 2018). At *DiV* 21 the FUS (P525L) neurons showed almost the same functional alterations as their counterparts of the WT-I line, compared to the respective WT: APs amplitude was reduced (supplementary Fig S2A), as well as glutamate (Fig. S2B) and glycine (Fig. S2C) responses; GABA responses (Fig. S2D) did not show any significant difference, differently to WT-I line.

These data highlight that the differences in the inhibitory response between WT and mutated neurons widen during maturation.

### Altered Expression of Neurotransmitter Receptors in hiPSC-Derived Spinal Neurons Carrying the FUS (P525L) Mutation

**Fig. 8:**
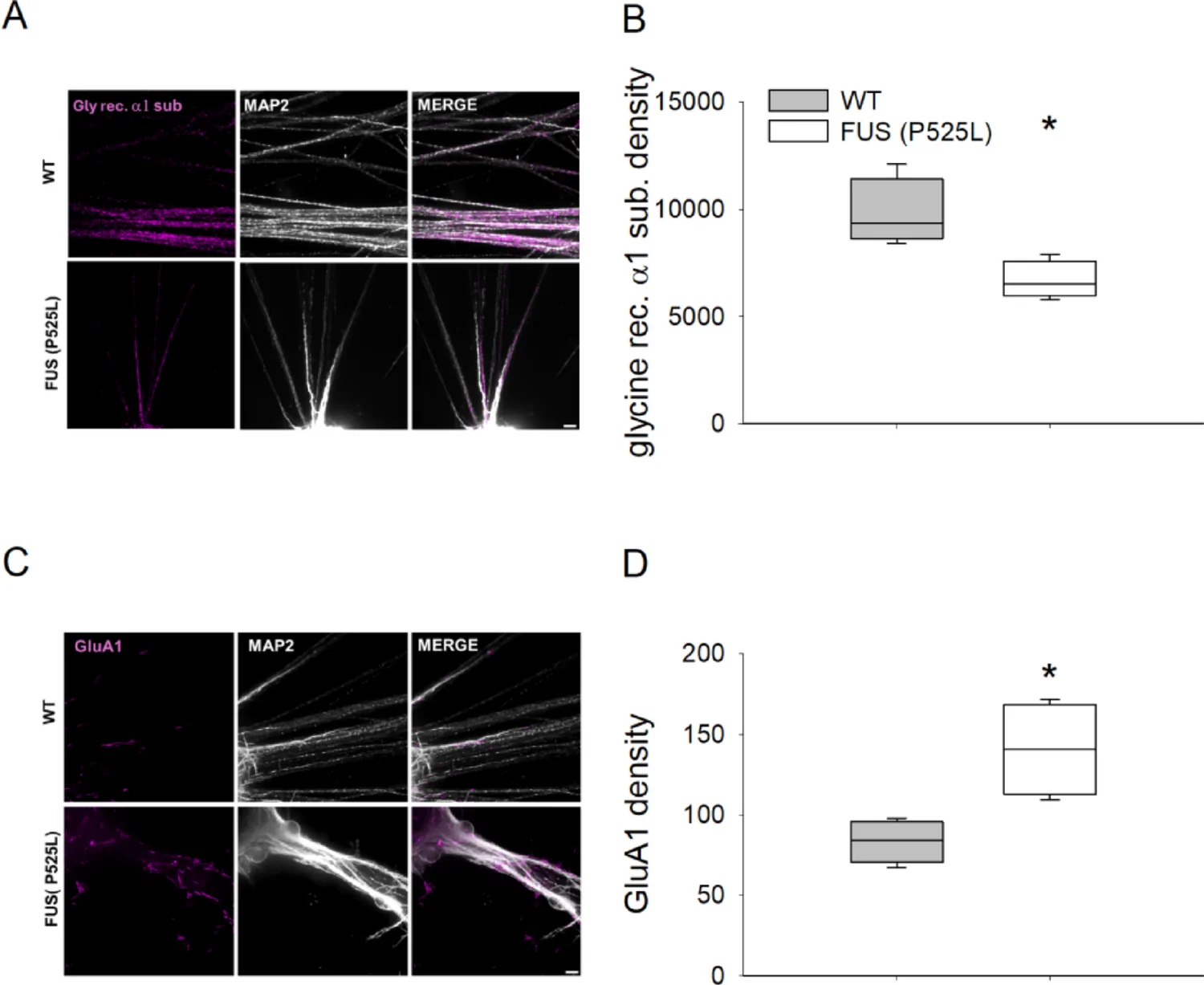
hiPSC-derived FUS (P525L) spinal neurons express a lower amount of glycine receptor α1 subunit and a higher amount of AMPA receptor GluR1 subunit.**A**, representative images of GLRA1 and MAP2 expression in hiPSCs-derived neurons at DiV 21. **B**, quantitative analysis of GLRA1 density normalized on MAP2 expression (*n* = 4 independent differentiation batches; *: F_1,6_ = 11.694 *p* = 0.014). **C**, representative images of GLUR1 and MAP2 expression in hiPSCs-derived spinal neurons at DiV 21. **D**, quantitative analysis of GLUR1 density normalized on MAP2 expression (*n* = 4 independent differentiation batches; **: *p* = 0.029). Scale bar = 10 µM. Data were obtained from hiPSC-derived spinal neurons of the WT I cell line. Significance was determined using a one-way ANOVA and attested on a p value *p* < 0.05

**Fig. 9:**
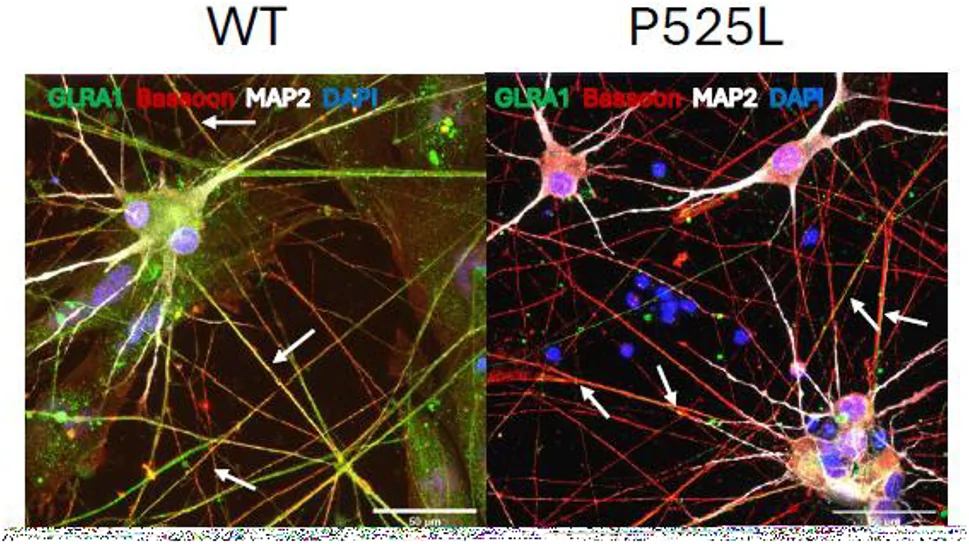
Colocalization of glycine receptor α1 subunit and Bassoon. Immunostaining analysis of spinal neurons culture at day 21 of differentiation. Bassoon (red) is a presynaptic marker; MAP2 is used as a neuronal markers. DAPI (blue) was used for nuclear staining. Arrows indicate colocalization of GLRA1 and Bassoon, revealing synaptic localization for GLRA1

Given the altered response to neurotransmitters observed in FUS (P525L) neurons, we investigated whether these differences could be attributed to changes in the expression of specific neurotransmitter receptors. The abundance of glycine receptor α1 and the AMPA-type glutamate receptor subunit 1 (GluA1) was measured by immunofluorescence analysis at DiV 21. Receptor puncta density was quantified and normalized to MAP2-positive area. FUS (P525L) neurons exhibited reduced expression compared to their isogenic WT controls, suggesting that the decreased glycine response may result from lower levels of glycinergic receptors (Fig. 8A, B). Conversely, GluA1 puncta density was significantly increased in the mutant spinal neurons compared to WT (Fig. 8C, D). Taken together, our data suggests that the altered neurotransmission observed in hiPSC-derived neurons carrying the FUS (P525L) mutation may be linked to the altered expression of key neurotransmitter receptors. Interestingly, glycine receptor α1 subunit immunofluorescence colocalized with Bassoon, a presynaptic marker, indicating a synaptic localization of the glycine receptor (Fig. 9).

## Discussion

ALS-related motoneuron malfunction and degeneration are widely described in literature (Marchand-Pauvert et al. 2019; Falgairolle and O’Donovan 2020; Jensen et al. 2020; Bączyk et al. 2022). In particular, familial ALS is caused by several mutations on a specific set of genes comprising *FUS* (Zou et al. 2017; Kamelgarn et al. 2018; Zhou et al. 2020). *FUS* encodes for a crucial RNA-binding protein that interacts with nascent transcripts and transcription machinery, preventing the formation of R-loops, which can alter transcriptional fidelity, playing a multifaceted role in cellular processes, particularly in RNA metabolism, transcription regulation, and autophagy (Thompson et al. 2022, 2023). FUS mutations, such as P525L, disrupt its nuclear localization and impair interactions with other proteins, leading to altered RNA metabolism (Garone et al. 2021). We have characterized the molecular and functional properties of hiPSC-derived spinal neurons during early maturation stages (*DiV 14*, *21*, *28*, *35*). Our neuronal cultures exhibited reduced pluripotency markers and activation of neuronal markers, as expected during differentiation, and the presence of CHAT, VGAT and GADD67. Interestingly, we observed the simultaneous presence of cholinergic and GABAergic markers in the same neuron, a finding already reported in several spinal neuronal populations (Kosaka et al. 1988; Gotts et al. 2016; Christoforidou et al. 2026). Previous electrophysiological studies on hiPSC-derived spinal neurons highlighted clear functional alterations, in particular an early increased excitability (Wainger et al. 2014; Yang et al. 2023) followed by a decreased of action potential output and synaptic activity, along with a dysfunction or loss of ion channels (Devlin et al. 2015). In our hands, both WT and FUS (P525L) neurons exhibit a general maturation during time, measured in terms of cell capacitance, membrane potential, synaptic activity, action potential properties, but mutated neurons display significant functional differences. Our data show a different time evolution of inhibitory and excitatory currents in WT and FUS (P5252L) neurons, with a larger decrease in inhibitory GABAergic and glycinergic currents in mutated cells, generating an altered excitatory/inhibitory balance which could lead to hyperexcitability and excitotoxicity. Previous evaluation of the cytopathological features of neuronal degeneration in the same lines used for the present work highlighted significantly increased and earlier apoptosis in FUS (P525L)-muscle co-cultures compared with FUS WT controls, along with impaired NMJ formation (Silvestri et al. 2024). Furthermore, FUS-mutated hiPSC-derived neurons exhibited increased glutamate-related excitotoxicity vulnerability (Shum et al. 2023). Neurodegenerative processes have been extensively studied in animal models of ALS: one notable example is the SOD1 (G93A) transgenic mouse, but also the models based on TDP-43 variants or forced mislocalisation (Cheng et al. 2024). These models have raised hypotheses about the origin of hyperexcitability in ALS: e.g. an imbalance in motoneurons and interneurons presence and transmission, which leads to an excitatory/inhibitory imbalance (Clark et al. 2017; Tessitore et al. 2026), or an aberrant localisation of TDP-43 in motoneurons (Reale et al. 2023).

Particularly interesting is the substantial reduction of the glycinergic response, observed for the first time, to our knowledge, in hiPSC-derived spinal neurons. This decrease is paralleled to a reduction of the expression of the glycine receptor α1 subunit and has been previously observed in human ALS patients and murine models of familial ALS with SOD1 mutation (Hayashi et al. 1981; Chang and Martin 2011). Hayashi and coworkers observed that glycinergic receptor binding was greatly reduced (16% of control value) in the anterior grey mater of ALS patients (Hayashi et al. 1981). The authors hypothesized that this reduction may be due to loss of large neurons in the anterior grey matter, but our data suggest that it could be due, at least in part, to a decrease of the number of glycine receptors in individual neurons. Moreover, the difference of inhibitory responses in WT and FUS (P525L) neurons widens during maturation, suggesting that this pathogenic variant may cause functional alterations not only in the earlier stages but also in later periods.

Our data highlight other functional differences. First, in mutated spinal neurons the membrane resting potential did not undergo the typical shift towards hyperpolarization in the latest stage, as already observed in other ALS-related hiPSC-derived neurons (Wainger et al. 2014). Second, we observed a strong reduction in the percentage of mutated neurons showing spontaneous synaptic activity compared to their WT counterparts, confirming the possibility of a slower or reduced maturation process. Last, coherently with a delayed maturation, early stages FUS (P525L) spinal neurons exhibited also lower APs amplitude and threshold, that revert during maturation. The relation between injected current and APs firing frequency was the same in the two groups at DiV 35, indicating that in mature neurons the mutation-related functional alteration does not extend to the APs machinery.

Glutamate-mediated excitotoxicity is a well-established pathological mechanism in ALS, and increased expression of GluA1 subunit and mGluRs (group I metabotropic glutamate receptors) have been reported in the spinal cords of SOD1 (G93A) mouse models (Zhao et al. 2008; Bonifacino et al. 2019). FUS protein also regulates the stability of GluA1 mRNA by binding to its mRNA, stabilizing it through poly (A) tail maintenance, thereby controlling its expression (Udagawa et al. 2015). FUS depletion has been linked to reduced GluA1 levels, affecting synaptic transmission and contributing to behavioral aberrations associated with ALS (Udagawa et al. 2015). In our experiments, in mutant neurons GluA1 subunit expression increased compared to their WT counterparts, in contrast with the reduction of glutamate-evoked currents, suggesting an impairment in the protein membrane insertion, folding, assembly or function.

The same electrophysiological experiments done at DiV 21 on a second independent hiPSC line (the T12.9) basically confirmed the observed functional alterations. Thus, our data indicate that the FUS (P525L) mutation causes a general reduction in the maturation rates and function of hiPSC-derived spinal neurons, with a relevant reduction in inhibitory signalling, which may produce a change in the excitatory/inhibitory balance, possibly linked to excitotoxicity and neurodegeneration.

## Supplementary Information

Below is the link to the electronic supplementary material.

Supplementary Material 1

## Data Availability

The datasets generated during and/or analysed during the current study are available in the IRCSS Neuromed repository, https://repository.neuromed.it/index.php/s/cNnmRWLApfmQmTt.
