# Supplementary material for: Human iPSC-Derived Spinal Neurons Carrying the ALS FUS (P525L) Mutation Exhibit Lower Response to Inhibitory Neurotransmitters
Source: Cell Mol Neurobiol. 2026 Jul 10;46:148. doi: 10.1007/s10571-026-01773-z (PMC13635062; doi:10.1007/s10571-026-01773-z)
Supplement: Supplementary file 1 [file 10571_2026_1773_MOESM1_ESM.docx]

***Human iPSC-derived spinal neurons carrying the ALS FUS (P525L) mutation exhibit lower response to inhibitory neurotransmitters***

Tiziano D’Andrea^1^, Maria Cristina Benedetti^2,3^, Valeria de Turris^3^, Alessandro Rosa^2,3^, Sergio Fucile^1,4^

^1^IRCCS Neuromed, Pozzilli (Is), Italy

^2^Department of Biology and Biotechnologies “Charles Darwin”, Sapienza University of Rome, Rome, Italy.

^3^Center for Life Nano- & Neuro-Science, Fondazione Istituto Italiano di Tecnologia (IIT), Rome, Italy.

^4^Department of Physiology and Pharmacology, Sapienza University of Rome, Rome, Italy.

***Molecular Neurobiology***

Corresponding author email: t.dandrea@uniroma1.it

**Supplementary Figure legends:**

**Fig. S1 FUS (P525L) mutation causes similar functional alterations in a different hiPSCs cell line.**

A, Amplitude (from threshold to peak) of APs evoked from WT (grey) and FUS (P525L) neurons (white) at DiV 21 (*: r = 5.968, p = 0.013). B, GABA current densities measured from WT and FUS (P525L) neurons at DiV. 21. C, glycine current densities measured from WT and FUS (P525L) neurons at DiV 21 (*: r = 11.518, p < 0.001). D, glutamate current densities measured from WT and FUS (P525L) neurons at DiV 21 (*: r = 8.782, p = 0.001). Data were obtained from iPSC-derived spinal neurons of the T 12.9 cell line, n _WT_ = 18, n _FUS(P525L)_ = 16. Significance was determined using a one-way ANOVA and attested on a p value *p* < 0.05.

**Fig. S2 TTX administration doesn’t alter FUS (P525L) mutation functional impairment in WT I cell line.**

A, glycine current densities measured from WT and FUS (P525L) neurons at DiV. 21 in presence of 100 nM TTX (*: r = 11.785 p < 0.001). B, GABA current densities measured from WT and FUS (P525L) neurons at DiV 21 in presence of 100 nM TTX (*: r = 9.346, p = 0.002). C, glutamate current densities measured from WT and FUS (P525L) neurons at DiV 21 in presence of 100 nM TTX (*: r = 6.836, p = 0.031). Data were obtained from iPSC-derived spinal neurons of the T 12.9 cell line, n _WT_ = 16, n _FUS(P525L)_ = 16. Significance was determined using a one-way ANOVA and attested on a p value *p* < 0.05.

*Figure S1:*

*Figure S2:*

*Figure S1:*
